# Supplementary material for: FOXO1 enhances CAR T cell stemness, metabolic fitness and efficacy
Source: Nature. 2024 Apr 10;629(8010):201–10. doi: 10.1038/s41586-024-07242-1 (PMC11062918; doi:10.1038/s41586-024-07242-1)
Supplement: Supplementary file 1 — Gating strategy for the analysis of mouse (a) and human (b) CAR T cells by flow cytometry. [file 41586_2024_7242_MOESM1_ESM.pdf]

nature portfolio

https://doi.org/10.1038/s41586-024-07242-1

Supplementary information

FOXO1 enhances CAR T cell stemness, metabolic fitness and efficacy

In the format provided by the authors and unedited

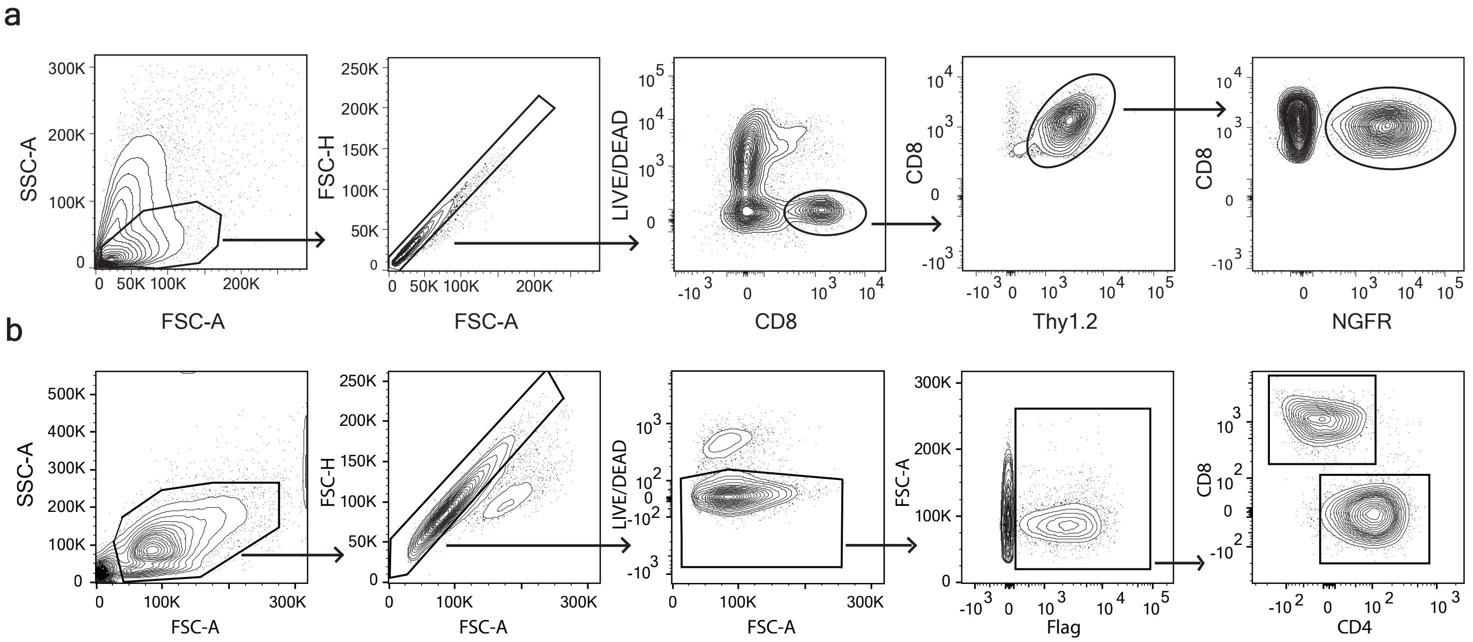

Supplementary Figure 1: Gating strategy for the analysis of mouse (**a**) and human (**b**) CAR T cells by flow cytometry. Cell doublets were excluded by gating cells on the diagonal of FSC-A/FSC-H. Dead cells were excluded by staining with LIVE/DEAD™ Fixable Yellow Dead Cell Stain (ThermoFisher). **a**, mouse $CD8^+$ CAR T cells were defined as Thy1.2$^+$ $CD8^+$NGFR$^+$. b, human CAR T cells were defined as Flag$^+$ and $CD4^+$ or $CD8^+$.
