# Supplementary material for: FOXO1 enhances CAR T cell stemness, metabolic fitness and efficacy
Source: Nature. 2024 Apr 10;629(8010):201–10. doi: 10.1038/s41586-024-07242-1 (PMC11062918; doi:10.1038/s41586-024-07242-1)
Supplement: Supplementary file 2 — Reporting Summary [file 41586_2024_7242_MOESM2_ESM.pdf]

nature portfolio

Corresponding author(s): Phillip K. Darcy, Paul A. Beavis, Junyun Lai

Last updated by author(s): Jan 19, 2024

Reporting Summary

Nature Portfolio wishes to improve the reproducibility of the work that we publish. This form provides structure for consistency and transparency in reporting. For further information on Nature Portfolio policies, see our Editorial Policies and the Editorial Policy Checklist.

Statistics

For all statistical analyses, confirm that the following items are present in the figure legend, table legend, main text, or Methods section.

n/a	Confirmed	
☐	☒	The exact sample size (n) for each experimental group/condition, given as a discrete number and unit of measurement
☐	☒	A statement on whether measurements were taken from distinct samples or whether the same sample was measured repeatedly
☐	☒	The statistical test(s) used AND whether they are one- or two-sided<br>*Only common tests should be described solely by name; describe more complex techniques in the Methods section.*
☐	☒	A description of all covariates tested
☐	☒	A description of any assumptions or corrections, such as tests of normality and adjustment for multiple comparisons
☐	☒	A full description of the statistical parameters including central tendency (e.g. means) or other basic estimates (e.g. regression coefficient) AND variation (e.g. standard deviation) or associated estimates of uncertainty (e.g. confidence intervals)
☐	☒	For null hypothesis testing, the test statistic (e.g. F, t, r) with confidence intervals, effect sizes, degrees of freedom and P value noted<br>*Give P values as exact values whenever suitable.*
☒	☐	For Bayesian analysis, information on the choice of priors and Markov chain Monte Carlo settings
☒	☐	For hierarchical and complex designs, identification of the appropriate level for tests and full reporting of outcomes
☒	☐	Estimates of effect sizes (e.g. Cohen's d, Pearson's r), indicating how they were calculated

Our web collection on statistics for biologists contains articles on many of the points above.

Software and code

Policy information about availability of computer code

Data collection	Flow cytometry: BD FacsDiva version 8 (FlowJo LLC)<br>Seahorse assay: Seahorse XFe24 Bioanalyser (Agilent)<br>RNA-seq: CASAVA v1.8.2, Cutadapt v2.1, FastQC v0.11.6, RNA-SeQC v1.1.8<br>ATAC-Seq: Bcl2fastq (v2.20)
Data analysis	Flow cytometry: FlowJo version 10 (FlowJo LLC)<br>Differentially expressed gene analysis: EdgeR (https://bioconductor.org/packages/release/bioc/html/edgeR.html) v4.01<br>Gene set enrichment analysis: Enrichr (http://amp.pharm.mssm.edu/Enrichr)<br>General: Microsoft Excel 2010<br>Statistical analysis and data presentation: Graphpad Prism 9<br>Gene expression analysis: featureCounts, Rsubread 2.10.5; heatmaps: pheatmap R package v1.0.12, HISAT2 v 2.0.4<br>ATAC-Seq: MACS2 (v2.1.1), Genrich (v0.6.0), Homer v4.11, ChIPseeker (v1.8.6), IGV v2.7.0, findMotifsGenome, chromVAR

For manuscripts utilizing custom algorithms or software that are central to the research but not yet described in published literature, software must be made available to editors and reviewers. We strongly encourage code deposition in a community repository (e.g. GitHub). See the Nature Portfolio guidelines for submitting code & software for further information.

Data

Policy information about availability of data

All manuscripts must include a data availability statement. This statement should provide the following information, where applicable:

- Accession codes, unique identifiers, or web links for publicly available datasets
- A description of any restrictions on data availability
- For clinical datasets or third party data, please ensure that the statement adheres to our policy

Data availability statement has been provided in the manuscript as below. Additional raw data are available from the corresponding authors upon request.

Data availability Statement
The RNA-Sequencing and ATAC-Sequencing data that supports the findings of this study have been deposited in GEO NCBI under the accession code GSE225527 that contains the subseries GSE225521, GSE225522, GSE225523 and GSE225526. Reference gene sets were obtained from the MsigDB library for Hallmarks (https://www.gsea-msigdb.org/gsea/msigdb/human/collections.jsp), KEGG (https://www.genome.jp/kegg/kegg1.html), CHEA dataset 47, 48, 49, 50, or based upon previously published analyses of glycolysis signature 23, single-cell RNA sequencing derived T cell clusters in patients 11. Reference databases used are hg38 (https://www.ncbi.nlm.nih.gov/datasets/genome/GCF_000001405.26/), mm10 (Mus musculus genome assembly GRCm38 - NCBI - NLM (nih.gov)) and hg19 (https://www.ncbi.nlm.nih.gov/datasets/genome/GCF_000001405.13/). Source data are available within the paper (Microsoft® Excel® for Microsoft 365), supplementary information or available upon request from the authors.

Human research participants

Policy information about studies involving human research participants and Sex and Gender in Research.

Reporting on sex and gender	N/A
Population characteristics	Patients (aged >18 years) and had advanced solid tumours (local incurable or metastatic disease) and satisfied the eligibility criteria.
Recruitment	Patients were accrued onto the phase I clinical trial LeYPh1-02, trial number NCT03851146) following assessment of eligibility criteria as outlined on https://www.clinicaltrials.gov/ct2/show/study/NCT03851146. Eligibility criteria included advanced solid tumours which were (>10% LeY+ tumour cells positive) by IHC.
Ethics oversight	Ethics for the study was approved by the Peter MacCallum Cancer Centre Human Research Ethics committee.

Note that full information on the approval of the study protocol must also be provided in the manuscript.

Field-specific reporting

Please select the one below that is the best fit for your research. If you are not sure, read the appropriate sections before making your selection.

☒ Life sciences ☐ Behavioural & social sciences ☐ Ecological, evolutionary & environmental sciences

For a reference copy of the document with all sections, see nature.com/documents/nr-reporting-summary-flat.pdf

Life sciences study design

All studies must disclose on these points even when the disclosure is negative.

Sample size	Experiments were performed with sufficient power to achieve statistical significance based upon an effect size of 30%, which would have been deemed clinically significant. All therapeutic experiments were performed with a minimum of 3 mice per group.
Data exclusions	No data were excluded from the manuscript.
Replication	All experiments were replicated in at least 2 independent experiments.
Randomization	Mice were randomized prior to treatment according to tumor size to ensure all groups had equivalent tumor burden prior to therapy. Groups were age and sex matched. Randomization did not apply to in vitro studies because experiments were conducted with a common source of biological material e.g. the same PBMCs.
Blinding	Data was not blinded. The same investigators performed and analyzed experiments and so blinding was not possible.

Reporting for specific materials, systems and methods

We require information from authors about some types of materials, experimental systems and methods used in many studies. Here, indicate whether each material, system or method listed is relevant to your study. If you are not sure if a list item applies to your research, read the appropriate section before selecting a response.

Materials & experimental systems

n/a	Involved in the study	
☐	☒	Antibodies
☐	☒	Eukaryotic cell lines
☒	☐	Palaeontology and archaeology
☐	☒	Animals and other organisms
☒	☐	Clinical data
☒	☐	Dual use research of concern

Methods

n/a	Involved in the study	
☒	☐	ChIP-seq
☐	☒	Flow cytometry
☒	☐	MRI-based neuroimaging

Antibodies

Antibodies used: All antibodies used in the study were obtained from commercial suppliers (BD Pharmigen, Cell Signaling, eBioscience, Invitrogen, Thermo Scientific or Biolegend). A list of relevant information on antibodies is provided in the Supplementary Table

Validation: All antibodies were validated by the supplier. Relevant information can be found in the Supplementary Table

Eukaryotic cell lines

Policy information about cell lines and Sex and Gender in Research

Cell line source(s): The murine colon adenocarcinoma MC38-Her2 cell line was generated from cells obtained from Dr. Jeff Schlom (National Institute of Health, Maryland, USA). The mouse breast carcinoma E0771-Her2 cell line were generated from cells obtained from Prof. Robin Anderson (Olivia Newton-John Cancer Centre, Victoria, Australia). These cell lines were not obtained from a commercial source. PA317, GP+e86 and HEK293T, OVCAR-3 and MCF7 tumor cell lines were obtained from the American Type Culture Collection.

Authentication: Cell lines were not authenticated but were uitiized within 10 passages of a master stock

Mycoplasma contamination: All lines were tested negative for mycoplasma contamination

Commonly misidentified lines (See ICLAC register): None of the cell lines are listed on the ICLAC database

Animals and other research organisms

Policy information about studies involving animals; ARRIVE guidelines recommended for reporting animal research, and Sex and Gender in Research

Laboratory animals: C57BL/6 mice, NSG and C57BL/6 human-Her2 (hHer2) transgenic mice were utilized where indicated. Mice were used between 6-16 weeks of age.

Housing was as follows;
Cage type – Allentown IVC
Bedding – irradiated corncob
Diet – Barastoc irradiated commercial rat and mouse pellets
Light cycle – 10 hour dark/14 hour light with a half hour sunrise and a half hour sunset period
Ambient temperature is set at 20oC +/- 1degree
Relative humidity is 40%

Wild animals: This study did not involve wild animals.

Reporting on sex: Studies utilizing E0771-Her2 and OVCAR-3 cells were performed in female mice. Studies using MC38-Her2 were performed in male mice.

Field-collected samples: No field collected samples were used in the study.

Ethics oversight: Ethics oversight was performed by the Peter MacCallum Cancer Centre Animal Experimentation Ethics Committee (AEEC) at the Peter MacCallum Cancer Centre.

Note that full information on the approval of the study protocol must also be provided in the manuscript.

Flow Cytometry

Plots

Confirm that:

☒ The axis labels state the marker and fluorochrome used (e.g. CD4-FITC).

☒ The axis scales are clearly visible. Include numbers along axes only for bottom left plot of group (a 'group' is an analysis of identical markers).

☒ All plots are contour plots with outliers or pseudocolor plots.

☒ A numerical value for number of cells or percentage (with statistics) is provided.

Methodology

Sample preparation	Blood was collected via submandibular or retroorbital bleed into tubes containing EDTA prior to euthanasia. Blood and spleen samples were treated twice or once respectively with ACK lysis buffer before staining for flow cytometry. Tumors were digested in SAFC DMEM media with 0.01 mg/mL DNase (Sigma Aldrich) and 1 mg/mL type IV collagenase for 30 minutes at 37oC. Following digestion, tumor samples were filtered twice through a 70 µm filter to create a single cell suspension and resuspended in Fc block prior to staining for analysis by flow cytometry.
Instrument	FACS data were obtained on a BD FACS Symphony A5 and BD LSRFortessa X-20 from the Peter MacCallum Cancer Centre flow cytometry core facility.
Software	Data was analyzed using Flowjo software 10.8.1
Cell population abundance	For experiments where cells with FACS sorted, re-analysis was performed to confirm sort purity
Gating strategy	FSC/SSC gate was first used to gate on the morphology of leukocytes. A singlet gates was next used (FSC-A vs FSC-h) to exclude doublets followed by a viability gate (Fixable Yellow or DAPI) to excluded dead cells. CAR T cells were identified using a T cell lineage marker (Thy1.2 or CD3) and transduction marker (mCherry, NGFR or FLAG).

☒ Tick this box to confirm that a figure exemplifying the gating strategy is provided in the Supplementary Information.
